# Thermal, Rheological, Structural and Adhesive Properties of Wheat Starch Gels with Different Potassium Alum Contents

**DOI:** 10.3390/molecules28186670

**Published:** 2023-09-17

**Authors:** Haibo Zhao, Hongbin Zhang, Qiang Xu, Hongdong Zhang, Yuliang Yang

**Affiliations:** 1 Institute for Preservation and Conservation of Chinese Ancient Books, Fudan University Library, Fudan University, Shanghai 200433, China; 2 State Key Laboratory of Molecular Engineering of Polymers, Department of Macromolecular Science, Fudan University, Shanghai 200433, China

**Keywords:** wheat starch, potassium alum, adhesive

## Abstract

Wheat starch (WS) is a common adhesive material used in mounting of calligraphy and paintings. Potassium alum (PA) has indeed been used for many centuries to modify the physicochemical properties of starch. Thermal analysis revealed that the presence of PA led to an increase in the gelatinization temperature and enthalpy of the starch gels. The leached amylose and the swelling power of the starch gels exhibited a maximum at the ratio of 100:6.0 (WS:PA, *w*/*w*). The rheological properties of starch gels were consistent with changes in the swelling power of starch granules. SEM observations confirmed that the gel structure became more regular, and the holes grew larger with the addition of PA below the ratio of 100:6.0 (WS:PA, *w*/*w*). The short-range molecular order in the starch gels was enhanced by the addition of PA, confirmed by FT-IR analysis. Mechanical experiments demonstrated that the binding strength of the starch gels increased with higher PA concentrations and decreased significantly after the aging process. TGA results revealed that PA promoted the acid degradation of starch molecules. This study provides a detailed guide for the preparation of starch-based adhesive and its applications in paper conservation.

## 1. Introduction

Mounting of calligraphy and paintings is an ancient Chinese art form that has been practiced for over a millennium, serving as a means to conserve and preserve these exquisite works. During this process, a crucial element employed as an adhesive is a meticulously prepared starch gel derived from wheat starch [1]. Wheat starch is obtained by carefully washing the dough of wheat flour to remove gluten [2]. After the gelatinization process of starch, starch gels have the ability to form hydrogen bonds with cellulose fibers present in artworks [3]. This bonding mechanism is crucial in ensuring the longevity and stability of the artwork. However, the naturally derived starch gel has certain limitations, including relatively low viscosity and easy retrogradation [4,5]. Researchers have extensively investigated chemical, physical, and enzymatic modifications as means to overcome challenges associated with retrogradation and enhance the physicochemical properties of starch gels [6]. Among these approaches, physical modification techniques have gained considerable popularity due to their cost-effectiveness and minimal environmental impact [7]. Chinese restorers have ingeniously discovered that blending wheat starch with potassium alum (PA, KAl(SO_4_)_2_·12H_2_O) yields a significant augmentation in the apparent viscosity and binding strength of starch gels as adhesive [8,9]. By embracing these innovative practices, the art of mounting calligraphy and paintings continues to thrive.

PA has already proven its significant effects on the properties of starch-based gels [10,11]. For instance, Li et al. demonstrated that PA could improve the tensile properties of potato starch dough and reduced breakage of the starch hydrogels [12]. Another study by Li et al. found that PA enhanced the stability of potato starch molecules, inhibited gelatinization, prevented starch molecule leakage, increased bound water content, and significantly enhanced the storage modulus [13]. Furthermore, Wang et al. reported that the effects of salts on the pasting and retrogradation of maize starch was in accord with the Hofmeister series [14]. In detail, potassium (K^+^) and sulfate (SO_4_^2−^), as salting-out ions, were found to lower the swelling power and solubility of starch. In contrast, salting-in ion could increase these properties [15,16,17,18]. Moreover, Margit et al. observed that divalent cations such as calcium (Ca^2+^) and magnesium (Mg^2+^) had a greater impact in reducing starch recrystallization rate compared to monovalent cations [19]. PA consists of three ions, including two salting-out ions (K^+^ and SO_4_^2−^) and one multivalent cation (Al^3+^). As a result, the influence of PA on starch mixtures encompasses the comprehensive effects of these three ions working together.

The use of PA blended with wheat starch suspension to enhance the apparent viscosity and adhesive strength of starch gels has been a practice for many centuries. The ratios of WS:PA (*w*/*w*) used in restoration practices depend on the size of the works being restored. In general, the ratios range from 100:2 to 100:20 (WS:PA, *w*/*w*). However, the impact of PA on the apparent viscosity of wheat starch gels and its relationship with the binding capacity of the gel have not been thoroughly investigated. Furthermore, PA is an acid salt that has the potential to cause acid degradation and color change in paper relics, which has raised concerns among restorers [20,21]. Therefore, we aim to study the effects of PA on the thermal, rheological, and structural properties of wheat starch gels. Additionally, the relationship between adhesive strength and apparent viscosity of the starch gel was carefully determined. To evaluate the overall effects of PA on relics, including mechanical properties and color difference, aging experiments were conducted on paper samples bonded using WS-PA gels with various ratios. Through this comprehensive research, we aim to gain a deeper understanding of how PA influences the physicochemical properties of what starch gels and the influence of WS-PA gels on the conservation and preservation of precious paper relics.

## 2. Results

### 2.1. Thermal Properties

**Figure 1:**
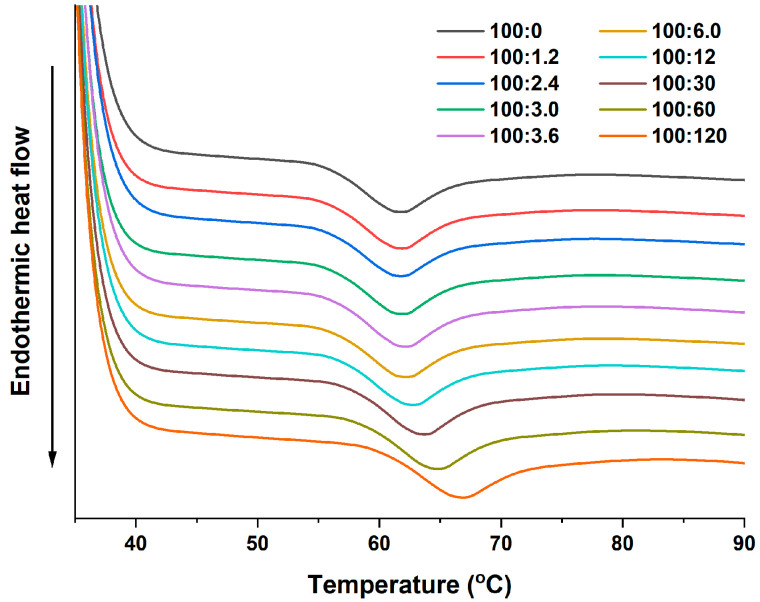
Gelatinization thermograms of WS and WS–PA mixtures.

The gelatinization curves of the WS and WS-PA mixtures were analyzed by DSC, and the pasting parameters were calculated using specialized software. Figure 1 illustrates the gelatinization process, while Table 1 presents the values of the pasting parameters. As depicted in Figure 1, the addition of PA in the starch mixtures can delay the pasting process, and the extent of this delay is influenced by the concentration of PA. The pasting temperature always increases with the addition of PA. These temperature changes are related to the transition of the ordered structure in starch granules to amorphous structure during gelatinization [22]. Two reasons can explain this phenomenon. Firstly, the ions in PA can interact with water molecules and decrease the amount of free water available relative to starch, thereby enhancing the stability of the starch granules [13,23]. Secondly, the Al^3+^ ions present in PA can combine with water to form positively charged hydrated forms such as Al(OH)^2+^, Al_2_(OH)_2_^4+^, Al_3_(OH)_4_^5+^, and Al_13_O_4_(OH)_24_^7+^ [24]. These hydrated products may act as crosslinking points, interacting with starch molecules in the amorphous regions or on the surface of starch granules. This crosslinking effect inhibits granule expansion and delays its destruction [14]. The gelatinization enthalpy (ΔH) is a measure of the energy required for the gelatinization process of starch and primarily characterizes the loss of the double helix structure and the melting of the crystalline structure within the starch [25]. The presence of PA significantly increased the gelatinization enthalpy of the starch mixtures. For instance, the enthalpy of a sample with ratio of 100:120 (WS:PA, *w*/*w*) increases by 8.7% compared to the blank sample. This increase indicates that more energy is needed to disrupt the starch granules. These findings are consistent with results concluded by Chen et al., who reported that multivalent cations can enhance the stability of the granules [12].

### 2.2. Leached Amylose and Swelling Power

**Figure 2:**
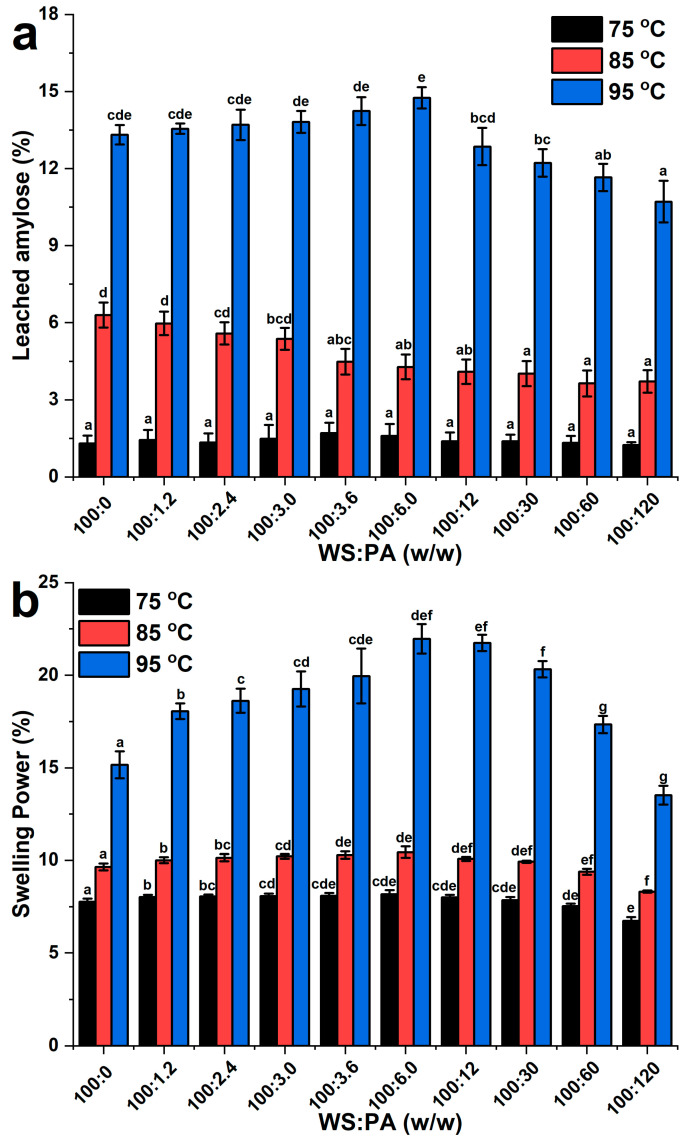
(**a**) The amount of leached amylose from WS and WS-PA gels at 75 °C, 85 °C and 95 °C, and (**b**) the swelling power of WS and WS-PA gels at 75 °C, 85 °C and 95 °C. The measurements were performed in triplicate. The mean ± SD values within the same column for each sample, followed by different lowercase letters, are significantly different (*p* < 0.05).

The leached amylose of samples is an important parameter that indicates the leaching of soluble amylose starch and segmental amylopectin from starch granules during gelatinization. Swelling power represents the water absorption capacity of starch granules [26]. Figure 2 shows the leached amylose and swelling power of WS and WS–PA gels at various ratios. As shown in Figure 2, both leached amylose and swelling power of WS and WS–PA gels increase with higher temperature [27]. The higher temperature facilitates the disintegration of starch granules and the disruption of the crystalline structure, leading to the leaching of amylose and the breakage of the double helix structure of amylopectin. Figure 2a demonstrates that the leached amylose of WS–PA mixtures at 95 °C increases with the addition of PA below the ratio of 100:6.0 (WS:PA, *w*/*w*) and decreases with the addition of PA above the ratio of 100:6.0 (WS:PA, *w*/*w*). Based on Figure 2b, the swelling power of the blends at 95 °C also reaches its maximum at a ratio of 100:6.0 (WS:PA, *w*/*w*), which is consistent with the results of leached amylose measurements. Different types of ions can significantly affect the pasting behavior of starch, in accordance with the Hofmeister series [28]. PA consists of K^+^, Al^3+^, and SO_4_^2−^ ions. Wang et al. discovered that SO_4_^2−^ and K^+^ ions, as salting-out agents, could improve the stability of starch granules [15]. Conversely, Al^3+^ ions, as multivalent cation, could bind with water molecules to form hydroxyl coordination compounds (Al(OH)_x_(H_2_O)_y_^n^) which may promote the disintegration of starch granules [24]. The results indicate that when the addition of PA is below the ratio of 100:6.0 (WS:PA, *w*/*w*), the PA could promote the disintegration of starch granules, with Al^3+^ ions playing a dominant role. However, when the addition of PA is above the ratio of 100:6.0 (WS:PA, *w*/*w*), the stability of starch granules is improved. There are two reasons to account for this phenomenon. Firstly, the SO_4_^2−^ and K^+^ ions play a dominant role as salt-out ions which could inhibit the breakage of starch granules. Secondly, the positively charged hydroxyl coordination compounds of Al^3+^ could act as cross-linking points through electrostatic interactions in the amorphous structure or on the surface of starch granules which inhibit the breakage of granules [14].

### 2.3. Rheological Measurements

#### 2.3.1. Dynamic Rheological Measurements

**Figure 3:**
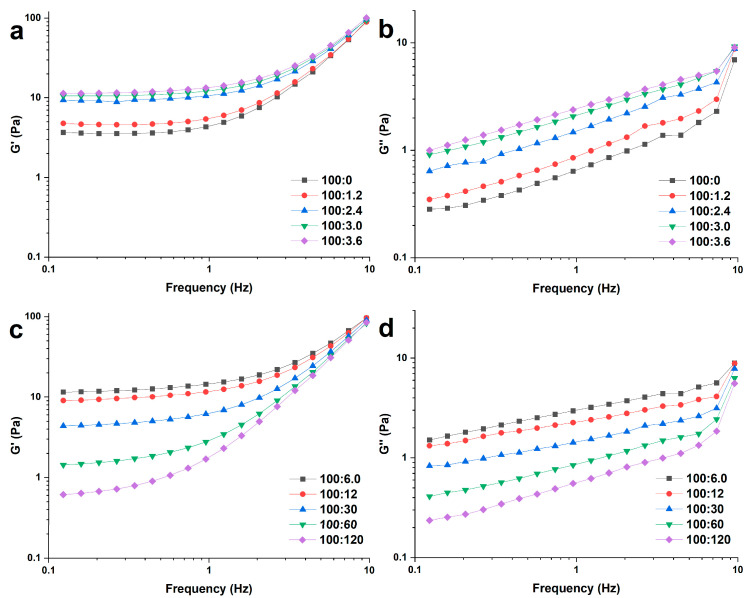
The dynamic rheological properties of WS and WS–PA gels. (**a**,**b**) Storage modulus (G′) and (**c**,**d**) loss modulus (G″) as a function of frequency at 25 °C and 1% strain.

The dynamic rheological properties of WS and WS–PA gels at different ratios of PA are analyzed and presented in Figure 3. The G’ and G’’ of all samples increase with frequency, and the G’ is consistently higher than the G’’ in all samples, indicating that the WS and WS–PA gels exhibit solid-like behavior [29]. In regards to the effect of PA concentration on the dynamic modulus, it is observed that when the addition of PA is below the ratio of 100:6.0 (WS:PA, *w*/*w*), the dynamic modulus increases with the presence of PA, as shown in Figure 3a. However, when the addition of PA is at higher concentrations, above the ratio of 100:6.0 (WS:PA, *w*/*w*), the dynamic modulus decreases as the PA concentration increases further. These findings are in agreement with the observed swelling power of the samples, suggesting that the WS–PA gel has the most compacted structure at the ratio of 100:6.0 (WS:PA, *w*/*w*). The results indicate that the breakage of granules reaches the maximum at this specific proportion and more-soluble starch leaching out from the granules participates in the network formation of WS–PA gels.

#### 2.3.2. Steady Rheological Measurements

**Figure 4:**
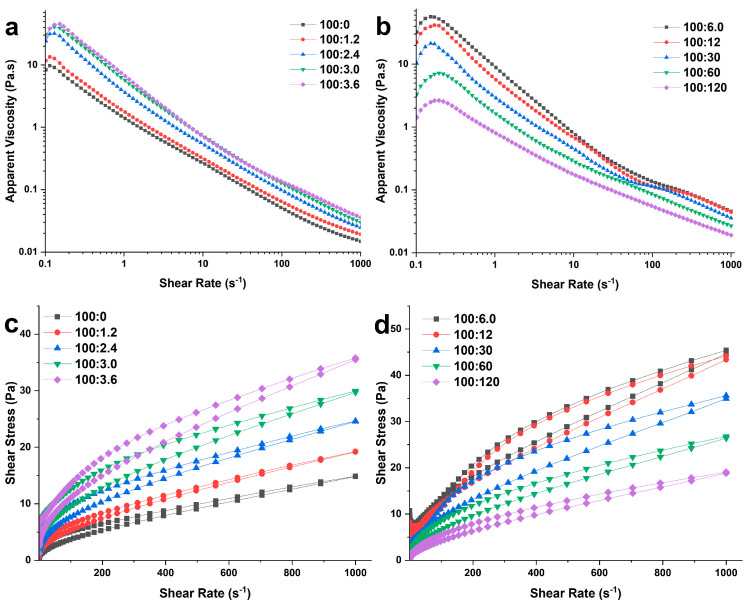
The steady rheological properties of WS and WS−PA gels. (**a**,**b**) Apparent viscosity and (**c**,**d**) shear stress as a function of shear rate.

Figure 4 presents the steady flow curves of WS and WS−PA gels, while Table 2 summarizes the fitting results of the steady flow curve data using the Herschel–Bulkley model. The R-squared values for all samples fitted by the Herschel–Bulkley model are above 0.964, indicating good model fit. All systems exhibit shear thinning behavior as the apparent viscosity of testing samples decreases with the increasing of shear rate. Additionally, the flow behavior index (n) values range from 0.51 to 0.77, which are significantly lower than 1. This suggests that both WS and WS−PA gels exhibit pseudoplastic fluid behavior with shear-thinning properties [30]. Moreover, it is observed that the apparent viscosity of WS−PA gels increases at low shear rates and reaches a maximum at a shear rate of 0.2 s^−1^. Firstly, this phenomenon can be attributed to the non-equilibration of the sample [31]. Secondly, when an external force is applied, the entanglement between the molecular chains of the material can gradually be disrupted, leading to relative displacement of the ordered structure under shear. This disruption of entanglements and the resulting relative displacement contribute to shear thickening. The addition of PA to WS gels has an impact on the apparent viscosity. The apparent viscosity increases with the augmentation ratios from 100:0 to 100:6.0 (WS:PA, *w*/*w*), but it then decreases as the ratios increase further from 100:6.0 to 100:120 (WS:PA, *w*/*w*). The consistency index (K) calculated by the Herschel–Bulkley model represents the viscosity of the gels. However, the behavior of K varied between ratios of 100:3.6, 100:6.0, and 100:12 (WS:PA, *w*/*w*). This decrease can be attributed to the destruction of the compact crosslinked structure of WS−PA gels at high shear rates, resulting in a significant decrease in viscosity and the lowest fitted value of K. The yield stress required to change the structure of the gels increases from 1.14 Pa to 7.74 Pa with the ratio from 100:0 to 100:6.0 (WS:PA, *w*/*w*). This indicates that more external force was required to alter the initial structure of starch gels at low addition concentrations of PA. However, the opposite trend is observed as the ratios increase from 100:6.0 to 100:120 (WS:PA, *w*/*w*) with the yield stress decreasing from 7.74 Pa to 0.37 Pa. This trend is consistent with the interpretation of the change in swelling power discussed in Section 2.2. Thixotropy is the ability of starch mixtures to recover their initial structure under external agitation or shear force [32]. The strength of thixotropy can be reflected by the area of the hysteretic loop, with a larger clockwise hysteretic loop indicating a more compact gel structure. The results indicate that the ratio at 100:6.0 (WS:PA, *w*/*w*) could result in obtaining the most compact structure of starch gels.

### 2.4. FT-IR

**Figure 5:**
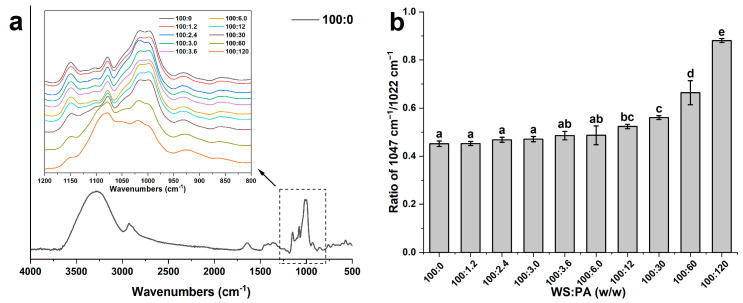
(**a**) FT-IR spectra of WS and WS−PA gels; (**b**) the ratio of absorption at 1047 cm^−1^ and 1022 cm^−1^. The measurements were performed in triplicate. The mean ± SD values within the same column for each sample, followed by different lowercase letters, are significantly different (*p* < 0.05).

The infrared spectra of WS and WS−PA gels are displayed in Figure 5a. Only one full IR spectrum of sample with ratio of 100:0 (WS:PA, *w*/*w*) is shown, and the others are displayed in Figure S1. The detailed IR spectrums of all samples in the wavenumber range of 1200–800 cm^−1^ are shown in the Figure 5a. All curves exhibit a noticeable absorption peak in the range of 3600–3000 cm^−1^, corresponding to the stretching vibrations of O-H bonds in starch [33]. The symmetric and antisymmetric stretching vibrations of -CH_2_ and -CH groups are detected at 3000–2800 cm^−1^ [29]. A minor absorption peak at 1550–1750 cm^−1^ indicates the bending vibration of water molecules within the sample. The fingerprint region of 1200–800 cm^−1^ is sensitive to conformation of the samples, particularly the absorption peaks at 1022 cm^−1^ and 1047 cm^−1^, which corresponds to the relative contents of random structures in the amorphous region and the ordered structures in the crystalline region, respectively. The ratio of the absorption peak intensity at 1047 cm^−1^ to 1022 cm^−1^ serves as a measure of starch crystallization degree. The results are presented in Figure 5b. It is observed that the ratio of the starch absorption peak at 1047 cm^−1^ to 1022 cm^−1^ increases with the addition of PA, further indicating that during the storage, PA within the system can effectively promote the ordered rearrangement of starch molecular chains.

### 2.5. SEM

**Figure 6:**
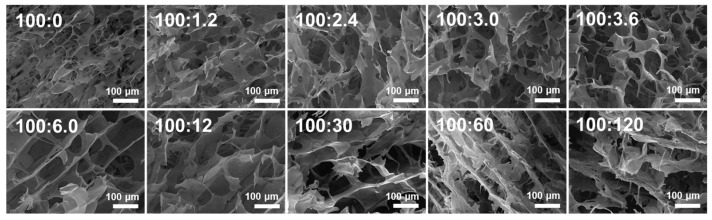
The SEM images of WS and WS−PA gels.

Figure 6 shows the SEM images of WS and WS−PA gels, magnified 500 times. The microstructure of WS and WS−PA gels resemble a honeycomb structure. However, compared to the control, the gels with addition of PA exhibit larger holes, and the size of the pores increases with higher PA concentrations. The pores in starch gels represent areas where water is distributed during storage. The larger pores and thicker walls in the gels indicate an enhancement of the entanglement force between starch chains generating the separation of water from the homogeneous gel phase [27]. As a result, the addition of PA facilitates the interaction between starch molecules. Furthermore, the structure becomes more uniform and regular and the size of pores grows larger with the increasing ratios of WS:PA from 100:0 to 100:6.0 (*w*/*w*). However, the opposite tendency can be observed at the higher ratios from 100:6.0 to 100:120 (*w*/*w*). The addition of PA promotes the breakage of starch granules below the ratios of 100:6.0 (WS:PA, *w*/*w*), and more starch molecules participate in the network formation of WS−PA gels. However, when the ratios are above 100:6.0 (WS:PA, *w*/*w*), the addition of PA inhibits the disintegration of granules. Hence, the structure becomes irregular and the size of pores grows smaller. Additionally, the lyophilized samples with the ratios above 100:30 (WS:PA, *w*/*w*) are brittle, which is perhaps related with the high crystallization degree of starch gels.

### 2.6. Thermogravimetry (TG)

**Figure 7:**
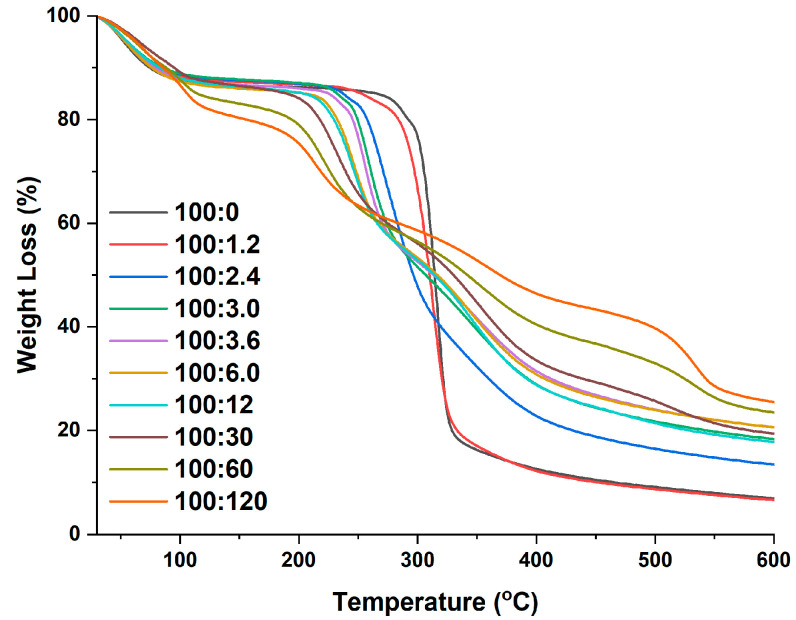
The TGA curves of WS and WS−PA gels.

Figure 7 shows the thermal degradation analysis of the lyophilized samples using TGA (thermogravimetric analysis). The thermal decomposition curves can be categorized into three main stages, providing information about the thermal stability of samples [34].The first stage (30 °C–150 °C) is related to the release of water and volatile substances [35]. The second stage (200 °C–335 °C) where the main weight loss occurs corresponds to the thermal decomposition of starch [36]. The third stage (400 °C–600 °C) is primarily attributed to the degradation of glycosidic bonds and further decomposition of polymer fragments. The addition of PA promotes the thermal degradation of starch molecules. Compared to the contrast group, the samples mixed with PA contain more moisture. This result can be attributed to the hydrolysis of Al^3+^, which generates various hydroxyl coordination compounds. These compounds can participate in the network formation of the starch gel. Additionally, a significant amount of hydrogen ions is generated during the hydrolysis process of Al^3+^, which facilitates the breakage of glycosidic bonds [37]. As a consequence, the temperature range of the second stage becomes narrower and lower when PA is added. Additionally, the addition of PA may also participate in the cross-linking reaction of starch fragments, inhibiting further decomposition of the starch. Hence, the third decomposition temperature to shift to a higher temperature. Moreover, the residual weight of the samples increases with the addition of PA, which can be attributed to the growing mass of PA in the samples.

### 2.7. Adhesive Properties

#### 2.7.1. Mechanical Properties

**Figure 8:**
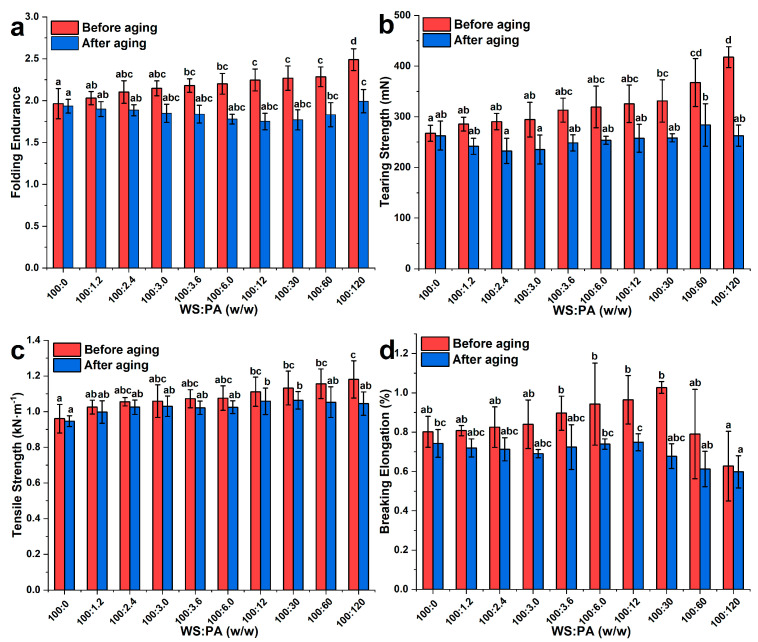
Effect of WS and WS−PA gels on the (**a**) tearing strength, (**b**) folding endurance, (**c**) tensile strength, and (**d**) breaking elongation of paper samples before or after aging experiments. The measurements were performed in triplicate. The mean ± SD values within the same column for each sample, followed by different lowercase letters, are significantly different (*p* < 0.05).

Figure 8 shows the mechanical properties of paper samples adhered by either WS or WS−PA gels. The mechanical testing results are directly related to the adhesive strength of the starch gels. There are two main factors that influence the mechanical properties of the testing samples. Firstly, the apparent viscosity and modulus of the starch gels play a significant role. These properties are closely related to the entanglement of starch chains and the ratio of ordered region to the amorphous structure [32]. The starch gels provide strength to the bonding layer between two paper samples. Secondly, the bonding strength between the starch and cellulose fibers on the paper surface is important. This factor is influenced by the permeability of the starch gels and the entanglement between the starch fragments and cellulose fibers. The addition of PA at ratios ranging from 100:0 to 100:120 (WS:PA, *w*/*w*) resulted in an increase in folding endurance from 1.96 ± 0.18 to 2.49 ± 0.13, tearing strength from 267.33 ± 15.81 (mN) to 417.66 ± 20.77 (mN), and tensile strength from 0.96 ± 0.08 (kN·m^−1^) to 1.18 ± 0.10 (kN·m^−1^). This indicates that the adhesive strength of starch gels was enhanced with the addition of PA. Interestingly, these results were not consistent with the changes observed in the apparent viscosity measured through steady rheology experiments. The reason for this phenomenon can be attributed to the positively charged hydroxyl coordination compounds of Al^3+^, which act as ion-bridges to connect the starch fragments and cellulose fibers of the paper [24,38]. These ion-bridges provide stronger bonding strength than other influencing factors. This explanation is further supported by the breaking elongation testing results shown in Figure 8d. The minimum elongation was observed at the ratio of 100:120 (WS:PA, *w*/*w*), indicating that the higher crosslinked density between cellulose fibers and starch fragments improved the hardness of the paper samples [39]. However, the mechanical properties of the testing samples greatly decreased after the aging experiments at 105 °C for 28 days, and the decline proportion increased with increasing PA addition concentrations. Specifically, the folding endurance, tearing strength, and tensile strength of the control group only decreased by 1.5%, 1.7%, and 1.4%, respectively. In contrast, the corresponding mechanical properties of paper samples adhered by WS−PA gels at a ratio of 100:120 (WS:PA, *w*/*w*) decreased by 19.9%, 37%, and 11.5%, respectively. The reason for this phenomenon can be related to the hydrolyzation of Al^3+^ ion, and a large number of H^+^ ions are generated during this process, which promotes the acid hydrolysis of glycosidic bonds in both starch chains and cellulose fibers. The decrease in mechanical properties of the testing samples after aging experiments highlights the significance of controlling the addition of PA to the starch gel in practical applications, as it can affect the durability and stability of the paper relics.

#### 2.7.2. Chromaticity Measurement

Table 3 presents the results of chromaticity measurements. After aging experiments, the color difference of the testing samples is noticeable. Specifically, as the concentrations of PA added into the starch gels increase, the surface of the paper samples becomes darker, redder, and more yellowish in appearance. This change in color can be attributed to the formation of chromophores resulting from the acid degradation of polysaccharides. Acidolysis poses a significant challenge for paper relics, particularly those made from mechanical wood pulp in the 20th century, which were often produced using alum-rosin sizing [40]. These paper relics are known to be brittle, yellowed, and have low pH values. The effect of aging on the color of paper samples is an important consideration in the restoration and preservation of paper relics, as it can impact their aesthetic appearance and historical value.

## 3. Materials and Methods

### 3.1. Materials

Wheat starch (amylose content: 24.3%, water content: 9.6%) was provided by Shanghai Yuanye Bio-Technology Co., Ltd., Shanghai, China. Potassium alum (PA) was provided by Sinopharm. Deionized water was used in this work. Xuan paper was obtained from China Xuan Paper Company Group, Jingxian, China.

### 3.2. WS−PA Systems Preparation

Different concentrations of PA solutions were prepared. Wheat starch was added into PA solutions to obtain 4% wheat starch solutions by oscillating. Samples with ten ratios of WS:PA (*w*/*w*), including 100:0, 100:1.2, 100:2.4, 100:3.0, 100:3.6, 100:6.0, 100:12, 100:30, 100:60, and 100:120, were labeled and used for further experiments. The starch gels were prepared by heating at 95 °C for 20 min under constant stirring. After heating, the resulting starch gels were stored at ambient temperature for 24 h.

### 3.3. Rheological Measurements

The rheological measurements were carried out by a rheometer (HAAKE MARS III, Thermofisher, Waltham, MA, USA). The experiments were conducted at a temperature of 25 °C using a parallel plate (35 mm diameter, 0.5 mm gap). Before testing, the samples were transferred onto the rheometer plate and equilibrated for 5 min. In order to analyze the dynamic rheological properties, the liner viscoelastic region was determined by a strain sweep measurement, and the strain for the frequency sweep experiment was set at 1% [41]. The frequency range was set from 0.1 to 10 Hz, and the dynamic modulus as functions of frequency was obtained. Steady shear experiments were conducted to measure the response of the samples to varying shear rates. The shear rate was increased from 0.1 s^−1^ to 1000 s^−1^ in 2 min and then decreased from 1000 s^−1^ back to 0.1 s^−1^ at the same speed. The apparent viscosity of WS−PA gels was recorded as a function of shear rate. To analyze the results of the steady shear tests, the data were fitted using the Herschel–Bulkley model, which is denoted by Equation (1) [42].

Τ = τ_0_ + Kx^n^ (1)

where τ_0_ represents the yield stress (Pa), τ represents shear stress (Pa), K is consistency index (Pa·s^n^), γ represents shear rate(s^−1^), and n is the flow behavior coefficient.

### 3.4. Leached Amylose and Swelling Power

The leached amylose and swelling power of WS and WS−PA gels were obtained according to the report of Chen et al. with a slight modification [43]. The slurries were prepared as per the descriptions of Section 3.2 and then the mixtures heated in an oscillating water bath at temperatures of 75 °C, 85 °C, and 95 °C for a duration of 30 min. After heating, the samples were immediately cooled to ambient temperature, and then the cooled samples were centrifuged at 4800 rpm for 30 min. The sediment was weighed and then dried to constant weight at 90 °C. The swelling power (%) was denoted as the ratio between the weight of the precipitate in its wet state and its dry weight. The amount of leached amylose in the supernatant were determined using the iodine colorimetric method as reported by Chrastil with a slight modification [44]. In detail, 0.05 mL of the supernatant obtained after centrifugation was added to 3 mL of 0.33 M NaOH in a water bath set at 95 °C for 30 min. After cooling to ambient temperature, the mixtures were centrifuged at 4800 rpm for 10 min. Then, 0.1 mL of the resulting solutions was mixed with 2.5 mL of trichloroacetic acid (0.5%, *v*/*v*) to adjust the pH of the solution to 5.0–6.0, and subsequently 0.01 N I_2_-KI (0.05 mL) was added. The mixture was reacted at room temperature for 30 min, and the absorbance of solution at 620 nm was measured using a spectrophotometer (TGem Plus, TIANGEN, Beijing, China). Amylose was used as the standard to calculate the dissolution amount of amylose.

### 3.5. DSC

The thermal analysis of wheat starch with different concentrations of PA was conducted using a DSC (DSC 250, TA Instruments, New Castle, DE, USA) under a N_2_ atmosphere [4]. To perform the analysis, 3 mg of wheat starch was mixed with 6 μL of various concentrations of PA solutions. This mixture was then hermetically sealed in an aluminum pan. To ensure full hydration, all samples were equilibrated at room temperature for 12 h. The temperature range for the analysis was set from 35 °C to 95 °C, and the heating rate was 10 °C/min. The DSC equipment software (TRIOS software version 5.4.0.300) was used to calculate several parameters of interest, including the onset temperature (To), peak temperature (Tp), conclude temperature (Tc), and the area of the main endothermic peak (J/g).

### 3.6. TGA

The WS−PA gels were prepared according to the methods in Section 3.2 and stored at −80 °C for 24 h. Subsequently, the samples were subjected to freeze–drying using a vacuum freeze dryer. To evaluate the thermal stability of the WS−PA samples, a thermal gravimetric analyzer (TGA, Mettler Toledo, Zurich, Switzerland) was employed. The experiments were carried out under a N_2_ atmosphere, and the heating rate was 20 °C/min. The temperature range was set from 30 °C to 600 °C. The weight loss of the samples was recorded as a function of temperature, indicating the decomposition or volatilization of different components contained in the samples [34,45].

### 3.7. ATR-FTIR

The lyophilized samples were obtained following the procedure described in Section 3.6. The measurements were conducted using an FT-IR instrument (Nicolet 6700, Thermofisher, Waltham, MA, USA) equipped with a diamond ATR accessory over the range from 4000 cm^−1^ to 500 cm^−1^. To collect the spectra, each sample was tested at a resolution of 4 cm^−1^, with an accumulation of 64 scans. One specific parameter of interest for evaluating the molecular order of starch gels is the ratio of absorbances at 1047 cm^−1^ and 1022 cm^−1^. This ratio provides an indication of the short-range molecular order present in the starch gels. By comparing the intensity of these two absorbance peaks, it is possible to evaluate the structural characteristics and organization of the starch gel [46].

### 3.8. SEM

The lyophilized samples were obtained following the procedure described in Section 3.6. The microstructure of the lyophilized samples was observed using SEM (VEGA 3 XMU, TESCAN, Brno, Czech) [47]. The accelerating beam voltage was set at 20 kV. To prepare the samples for SEM analysis, the cross-section of the lyophilized samples was coated with a layer of gold. After the gold coating, the samples were placed on the loading platform of the SEM using conductive adhesive tape. By utilizing SEM, high-resolution images of the sample’s surface can be obtained.

### 3.9. Paper Mechanical Properties Experiments

The starch gels were obtained as per the methods described in Section 3.2. The gels were coated evenly on one piece of paper (0.25 m × 0.25 m) by a coir scrub brush, and then another piece of paper was put on it and adhered together. After dried at 30 °C for 24 h, the paper samples were cut into different sizes for further mechanical properties experiments.

#### 3.9.1. Tensile Strength Experiment

The tensile strength was measured according to GB/T 12914-2008 at a constant elongation rate of 20 mm/min by a tensile strength tester (ZB-WLQ, Hangzhou Zhibang Automation Technology Co., Ltd., Hangzhou, China), and the size of paper samples was 15 cm × 0.75 cm.

#### 3.9.2. Folding Endurance Experiment

The folding endurance was measured according to GB/T 475-2008 by an MIT folding endurance tester (ZB-NZ135A, Hangzhou Zhibang Automation Technology Co., Ltd., Hangzhou, China). The size of paper samples was 15 cm × 1.5 cm, and the force was set at 4.98 N.

#### 3.9.3. Tearing Strength Experiment

The tearing strength was performed according to GB/T 455-2002 by a tearing strength tester (ZB-SL, Hangzhou Zhibang Automation Technology Co., Ltd., Hangzhou, China). The size of paper samples was 63 cm × 50 cm.

### 3.10. Color Evaluation

The color change of paper samples before and after the aging process was determined by the color coordinate values (Equation (2)) using an NR10QC colorimeter [48].

ΔE = [(ΔL*)2 + (Δa*)2 + (Δb*)2 ] ^1/2^ (2)

where “L*” represents brightness, “a*” represents red and green, and “b*” represents yellow and blue.

### 3.11. Statistical Analysis

All the measurements were conducted in triplicate to ensure accuracy and reliability of the results. The results were subjected to statistical analysis using SPSS statistical software (Version 20.0, SPSS Inc., Chicago, IL, USA). To assess the significance, analysis of variance (ANOVA) was performed on the data. In this case, Duncan’s test was used to further analyze the data, with a significance level set at *p* < 0.05. Additionally, the graphical representation of the data was created using Origin Pro software, specifically Version 8.0 by Stat-Ease Inc. (Minneapolis, MN, USA).

## 4. Conclusions

The addition of potassium alum to wheat starch gels was found to be an effective method for modifying their physicochemical properties. The experimental results show that addition of PA increases the gelatinization temperature and enthalpy value of the starch gels. However, it was observed that the leached amylose and swelling power of the starch gels reached a maximum at a certain ratio of WS:PA, specifically at the ratio of 100:6.0 (*w*/*w*). The rheological properties and SEM observations support this phenomenon. In practical applications, the addition of PA enhanced the bonding strength of the starch gels. The adhesive ability of the starch gels was improved with addition of PA. However, the addition of PA at high concentrations could have a negative effect on the surface color of paper samples. Therefore, it is advisable to carefully control the concentration of PA when adding it to starch gels. Keeping the addition of PA at low concentrations, specifically below the ratio of 100:6.0 (WS:PA, *w*/*w*), could help to mitigate the negative effects of PA on the degradation and color change of paper relics while still achieving the desired enhancements in rheological and adhesive properties.

## Figures and Tables

**Table 1:** Gelatinization temperatures and enthalpy of WS and WS–PA mixtures.

WS:PA (*w*/*w*)	To (°C)	Tp (°C)	Tc (°C)	ΔH (J/g)
100:0	55.98 ± 0.17 a	61.30 ± 0.21 a	65.91 ± 0.33 a	1.525 ± 0.025 a
100:1.2	56.08 ± 0.02 ab	61.38 ± 0.05 ab	66.00 ± 0.24 a	1.542 ± 0.005 ab
100:2.4	56.20 ± 0.05 bc	61.53 ± 0.10 ab	66.08 ± 0.19 ab	1.548 ± 0.024 ab
100:3.0	56.23 ± 0.03 bc	61.59 ± 0.05 ab	66.18 ± 0.20 ab	1.558 ± 0.073 ab
100:3.6	56.31 ± 0.10 cd	61.65 ± 0.07 b	66.27 ± 0.06 ab	1.571 ± 0.003 ab
100:6.0	56.45 ± 0.13 d	61.66 ± 0.10 b	66.42 ± 0.18 b	1.578 ± 0.064 ab
100:12	56.96 ± 0.07 e	62.34 ± 0.12 c	66.94 ± 0.12 c	1.582 ± 0.015 ab
100:30	57.75 ± 0.07 f	63.21 ± 0.18 d	67.87 ± 0.04 d	1.602 ± 0.065 bc
100:60	58.68 ± 0.12 g	64.20 ± 0.27 e	69.04 ± 0.26 e	1.649 ± 0.082 c
100:120	60.17 ± 0.19 h	66.20 ± 0.37 f	71.21 ± 0.36 f	1.657 ± 0.046 c

The measurements were performed in triplicate. The mean ± SD values within the same column for each sample, followed by different lowercase letters, are significantly different (*p* < 0.05).

**Table 2:** Steady flow parameters of WS and WS−PA gels.

WS:PA (*w*/*w*)	Up				Down				Hysteresis Loop
	τ_0_ (Pa)	K (Pa•s^n^)	n (-)	R^2^	τ_0_ (Pa)	K (Pa•s^n^)	n (-)	R^2^	
100:0	1.14 ± 0.24 ab	0.38 ± 0.12 bc	0.51 ± 0.04 a	0.992	0.55 ± 0.21 a	0.16 ± 0.08 a	0.65 ± 0.07 cd	0.998	668 ± 82 ab
100:1.2	1.48 ± 0.31 ab	0.41 ± 0.11 bcd	0.54 ± 0.03 ab	0.995	0.66 ± 0.09 a	0.31 ± 0.11 b	0.58 ± 0.03 ab	0.997	688 ± 97 a
100:2.4	3.34 ± 0.76 c	0.58 ± 0.04 def	0.51 ± 0.01 a	0.992	1.41 ± 0.05 b	0.49 ± 0.11 c	0.55 ± 0.02 a	0.998	1213 ± 483 b
100:3.0	4.97 ± 1.25 d	0.73 ± 0.11 e	0.51 ± 0.04 a	0.989	2.23 ± 0.45 cd	0.54 ± 0.11 c	0.56 ± 0.01 a	0.998	2069 ± 387 c
100:3.6	5.76 ± 0.44 d	0.48 ± 0.04 cde	0.61 ± 0.01 b	0.985	2.61 ± 0.22 de	0.57 ± 0.08 c	0.58 ± 0.01 ab	0.999	2160 ± 120 c
100:6.0	7.74 ± 1.34 e	0.21 ± 0.06 a	0.77 ± 0.05 c	0.964	2.85 ± 0.23 e	0.63 ± 0.05 c	0.61 ± 0.01 ab	0.998	2926 ± 292 d
100:12	5.29 ± 1.96 d	0.31 ± 0.16 ab	0.73 ± 0.09 c	0.974	2.07 ± 0.37 c	0.61 ± 0.07 c	0.61 ± 0.01 abc	0.998	2856 ± 214 d
100:30	2.19 ± 0.37 bc	0.65 ± 0.11 ef	0.58 ± 0.03 ab	0.989	1.18 ± 0.14 b	0.49 ± 0.01 c	0.61 ± 0.01 abc	0.998	2799 ± 230 d
100:60	0.91 ± 0.32 ab	0.62 ± 0.03 ef	0.54 ± 0.02 ab	0.998	0.58 ± 0.15 a	0.35 ± 0.03 b	0.62 ± 0.01 bc	0.998	1719 ± 146 c
100:120	0.37 ± 0.1 a	0.39 ± 0.06 bc	0.56 ± 0.02 ab	0.999	0.27 ± 0.02 a	0.18 ± 0.01 a	0.66 ± 0.01 d	0.999	1207 ± 176 b

The measurements were performed in triplicate. The mean ± SD values within the same column for each sample, followed by different lowercase letters, are significantly different (*p* < 0.05).

**Table 3:** The CIE Color Coordinates of paper samples coated with WS or WS−PA gels after aging at 105 °C for 28 days.

WS:PA (*w*/*w*)	L*	a*	b*	∆E
blank	91.81	−0.24	2.93	
100:0	90.67 ± 0.06 g	−0.2 ± 0.02 a	3.18 ± 0.07 a	1.17 ± 0.04 a
100:1.2	90.53 ± 0.04 g	−0.17 ± 0.04 ab	3.24 ± 0.09 a	1.32 ± 0.02 b
100:2.4	89.73 ± 0.11 f	−0.16 ± 0.07 ab	3.3 ± 0.11 ab	2.12 ± 0.09 c
100:3.0	89.68 ± 0.09 ef	−0.07 ± 0.04 bc	3.4 ± 0.12 b	2.19 ± 0.06 c
100:3.6	89.56 ± 0.08 de	−0.09 ± 0.05 c	3.74 ± 0.04 c	2.40 ± 0.06 d
100:6.0	89.48 ± 0.04 d	0.01 ± 0.03 d	4.05 ± 0.06 d	2.60 ± 0.01 e
100:12	89.19 ± 0.12 c	0.03 ± 0.03 de	4.3 ± 0.1 e	2.97 ± 0.06 f
100:30	89.08 ± 0.05 c	0.07 ± 0.02 def	6.93 ± 0.12 f	4.85 ± 0.07 g
100:60	88.9 ± 0.1 b	0.11 ± 0.07 ef	7.07 ± 0.05 f	5.07 ± 0.01 h
100:120	88.6 ± 0.11 a	0.13 ± 0.06 f	7.43 ± 0.07 g	5.54 ± 0.01 i

The measurements were performed in triplicate. The mean ± SD values within the same column for each sample, followed by different lowercase letters, are significantly different (*p* < 0.05).

## Data Availability

Data may be shared under request.

## Supplementary Materials

The following supporting information can be downloaded at: https://www.mdpi.com/article/10.3390/molecules28186670/s1, Figure S1: FT-IR spectra of WS and WS−PA gels.

## Sample Availability

Samples of the compounds are available from the authors.
