# Supplementary figures and images for: Thermal, Rheological, Structural and Adhesive Properties of Wheat Starch Gels with Different Potassium Alum Contents
Source: Molecules. 2023 Sep 17;28(18):6670. doi: 10.3390/molecules28186670 (PMC10534481; doi:10.3390/molecules28186670)

## Supplementary Materials

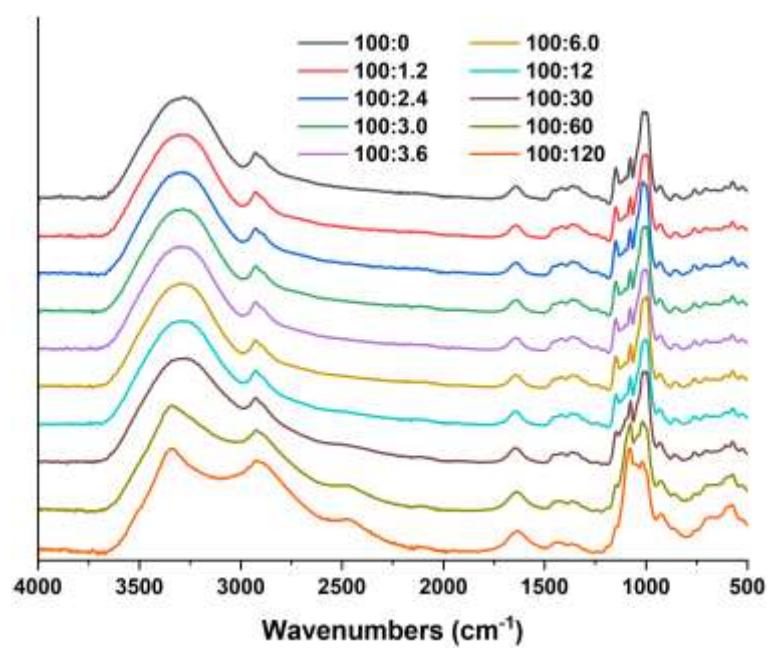

**Figure S1.** FT-IR spectra of WS and WS-PA gels.

Supplement: Supplementary file 1 [file molecules-28-06670-s001.zip › molecules-2595058-supplementary.pdf]
